# Novel Histopathologic and Ultrastructural Findings in Renal Allografts From Patients With Size and Age Mismatches: A Case Series

**DOI:** 10.7759/cureus.34510

**Published:** 2023-02-01

**Authors:** Cullen M Lilley, Maria M Picken

**Affiliations:** 1 Department of Pathology, Loyola University Medical Center, Maywood, USA

**Keywords:** ultrastructural pathology, renal pathology, age mismatch, size mismatch, kidney transplant

## Abstract

While reports on the long-term pathology in mismatched allografts have been focused on the donor and recipient body surface area, evidence is emerging to support donor-recipient age difference as an additional prognostic factor. Most reports are based on pediatric recipients receiving older/bigger allografts. Here, we describe three cases with age mismatch including two cases of adult patients receiving pediatric allografts and a third case of a younger patient receiving an allograft from an older donor exhibiting findings not described in extant literature. Each of these cases exhibits unique changes seen in mismatched donor-recipient size/age post-transplant pathology. These non-rejection changes should be suspected in cases of donor-recipient size/age mismatch. In cases of allograft function decline, a full biopsy workup, including electron microscopy, should be considered.

## Introduction

Nonrejection-related histopathologic and ultrastructural changes exhibited in renal allografts in the setting of acute/chronic allograft dysfunction are of utmost importance to transplant surgeons, nephrologists, pathologists, and transplant care providers. These changes not only provide the patient care team insights into the health of the allograft, but they also direct patient care. The spectrum of nonrejection histopathologic changes in kidney allografts is a testament to the improvements in human leukocyte antigen (HLA) identification techniques and immunosuppressive therapies available to patients [1]. Additionally, advancements in surgical techniques have led to more creative and complex donor-recipient situations [2-5]. These situations are most pronounced among pediatric populations, both as donors and recipients.

There is a growing body of literature surrounding the changes exhibited by allografts in the setting of donor-recipient size mismatch (i.e. the donor and recipient exhibit discrepancies in either body size or kidney size). Most research in this area has focused on the use of body surface area (BSA) or body mass index (BMI) to predict allograft outcomes and describe non-HLA donor mismatches [6-9]. However, there is growing evidence that age also plays a role in the pathologic changes observed in allografts in the post-transplant period that could also predict outcomes for kidney transplant recipients [7,10,11]. Based on the demographics of available kidney allografts, most of the studied age mismatches were informed by practices of older donors and younger recipients [4].

Based on the experiences from our institution, we identified three cases of age mismatch exhibiting unique histopathologic and/or ultrastructural changes not previously described. Of these three cases, we describe two cases of adult patients receiving a pediatric kidney allograft and one where a pediatric patient received an adult allograft. Here, we describe these three cases and discuss the potential repercussions of this limited case series on the field as a whole.

This article was previously presented as a meeting abstract at the 2022 American Society of Nephrology (ASN) Kidney Week Meeting on November 5, 2022.

## Case presentation

Case 1

**Figure 1:**
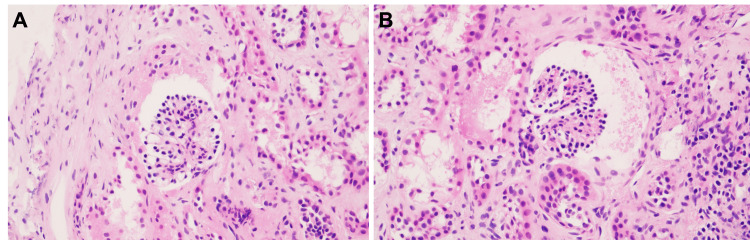
Representative Micrographs of Case 1 Histologic sections from case 1 stained with hematoxylin and eosin demonstrating persistent immature glomeruli; T-cell infiltrate was mild, < 15% (A, B). Original magnification 200x.

A 60-year-old female with a history of end-stage renal disease (ESRD) secondary to hypertensive nephrosclerosis (HNS) received bilateral kidneys en block from a five-day-old donor (Table 1). The patient's transplant course was complicated by perinephric hematoma, ureteral stent anastomosis blowout, and subsequent stent-related hydronephrosis. After surgical intervention and recovery, the patient developed hematuria with minor proteinuria and elevation in creatinine necessitating pathologic investigation. Biopsy of the allograft 96 days after transplantation revealed persistent immature glomeruli with cuboidal podocytes (WT-1 positive, not shown) (Figure 1). The biopsy also revealed focal PAS-positive Tamm-Horsfall glycoprotein in the interstitium but there were no vascular changes or thrombi present. Supportive care was given, and no further clinical intervention was required. The patient recovered with good renal function and allograft remains intact.

**Table 1:** Summary of Patient Cases Abbreviations: Bx, biopsy; UA, urinalysis; d, days; y, years; GBM, glomerular basement membrane; FSGS, focal segmental glomerulosclerosis

Case	Donor Age	Recipient Age	Gender Mismatch	Time to Bx	UA at time of Bx	Diagnosis	T-cell Infiltrate
1	5d	60y	?	96d	Hematuria	Immature Glomeruli	15%
2	14y	33y	Yes	65d	Oliguria with seroma hematuria	Thin GBM Disease	<10%
3	74y	21y	No	1057d	Proteinuria	FSGS-Alport-like	<5%

Case 2

**Figure 2:**
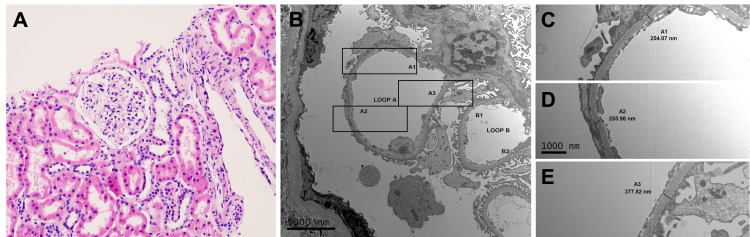
Histologic and Ultrastructural Micrographs of Case 2 Histologic (A) and ultrastructural (B-E) findings from case 2. By light microscopy the glomeruli were unremarkable and T-cell infiltrate was insignificant (<10%); hematoxylin and eosin stain, original magnification 200x (A). Ultrastructural analysis of the biopsy material demonstrated attenuation of the glomerular basement membrane (B). Representative measurements of the glomerular basement membrane thickness are shown in C-E; a majority of measurements fell below the lower end of normal (C-D) with rare segments reaching normal values (E).  Original magnification 5,000x (B) and 15,000x (C-E).

A 33-year-old male with a history of ESRD secondary to HNS received one kidney from a 14-year-old female donor (Table 1). The patient developed perinephric seroma, oliguria, and hematuria with associated elevation in creatinine necessitating pathologic investigation. Allograft biopsy 65 days after transplantation revealed segmental attenuation of the lamina densa with no electron-dense deposits (Figure 2). No changes to the patient’s immunosuppression or blood pressure regimens were required and the patient recovered with supportive care. Though renal function was excellent early after transplantation, the patient’s renal function did not ever reach the level of the other two allografts as determined by the estimated glomerular filtration rate (eGFR). On a subsequent biopsy one year later, segmental attenuation of the lamina densa persisted in addition to new cell-mediated allograft rejection consistent with Banff 4/1A rejection requiring alterations in immunosuppression [12].

Case 3

**Figure 3:**
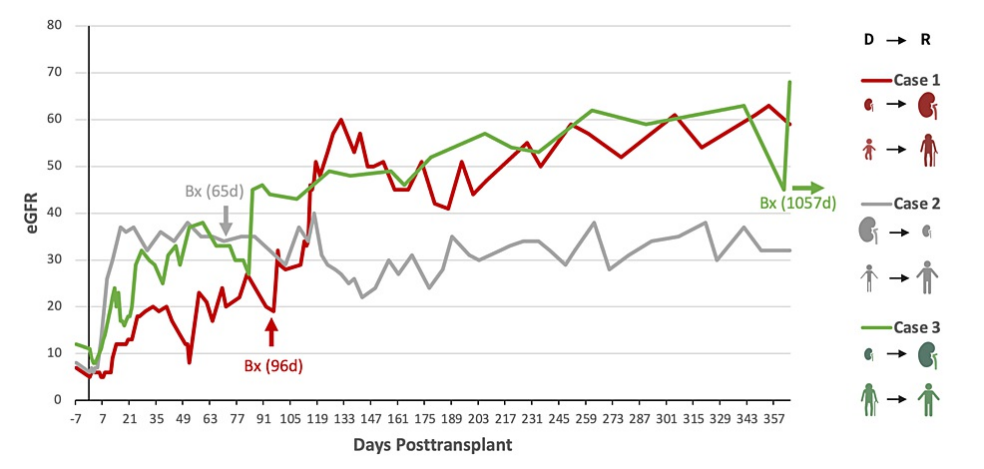
eGFR for Each Case in the First Year Post-transplantation This figure is a graphical representation of the three cases described with their associated estimated glomerular filtration rate (eGFR) results in the year following transplantation. The biopsy date (Bx) is also depicted on the graph. The right side of the figure depicts the type of mismatch being depicted (Left=donor (D), Right=recipient (R)) based on the difference in size of the kidneys (represented by the kidney icon) and the age difference (represented by the humanoid icons). Case 1 is depicted in red, case 2 is represented in grey, and case 3 is represented in green.

**Figure 4:**
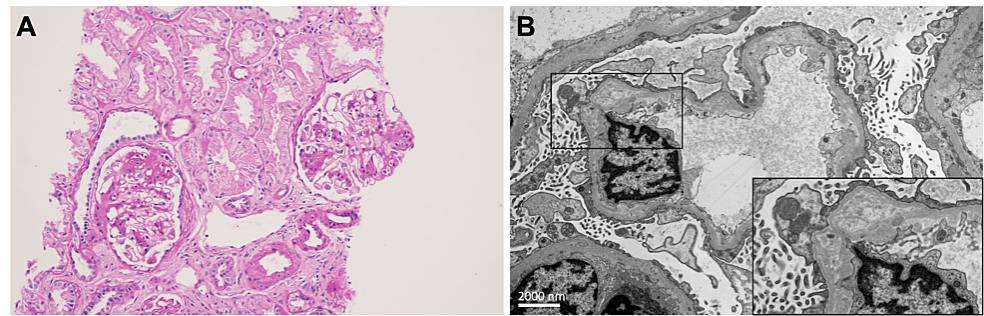
Histologic and Ultrastructural Micrographs of Case 3 Histologic (A) and ultrastructural (B & inset) findings from case 3 exhibiting focal segmental glomerulosclerosis evident on periodic-acid Schiff staining, original magnification 200x (A). On ultrastructural analysis, diffuse podocyte injury with podocyte effacement and microvillous transformation as well as focal areas of subendothelial space expansion and glomerular basement membrane multilayering and Alport-like changes (B & inset). Original magnification 7,000x and 15,000x (inset)

A 21-year-old female with ESRD of an unknown etiology received en block bilateral kidneys from a 74-year-old female donor (Table 1). Though both donor and recipient BMIs were in the "normal weight" range, the recipient BSA was likely much smaller, even though donor BSA was not available, because of the patient's height and young age at the time of transplantation. Post-surgery one allograft required nephrectomy owing to hemorrhagic necrosis; however, after this complication, the patient’s allograft experienced a predictable improvement in renal function in the year posttransplantation (Figure 3). 1057 days after transplantation, non-nephrotic-range proteinuria developed, and allograft biopsy showed focal and segmental glomerulosclerosis (Figure 4A) with podocyte effacement, microvillous formation, and glomerular basement membrane multilayering with Alport-like changes (Figure 4B). At the time of the biopsy, she was found to have a urinary tract infection and acute kidney injury. She was treated and discharged with slight renal allograft improvement. Unfortunately, this patient’s renal function continued to decline despite supportive measures, and she was placed on the transplant list.

None of these patients exhibited T-cell or antibody-mediated rejection evident by minimal to mild T-cell infiltrate on biopsy and negative donor-specific antibodies (DSA) and C4d stain; immunostain for polyomavirus were negative and all other immunofluorescence stains were also negative for immune complex glomerulonephritis.

## Discussion

In kidney allograft recipients, an acute decline in allograft function is worrisome and the etiologies for such a decline are broad including rejection and infection; however, in patients with a known donor-recipient size mismatch, other subtle structural changes to glomerular structures should be considered. In this limited case series, we described three unique donor-recipient kidney transplant scenarios all of which exhibited distinct histologic/ultrastructural findings and clinical outcomes.

Additionally, this case series explores scenarios that are not common in clinical practice, like case 1. Prior studies exploring clinicopathologic findings in age/size mismatched donor-recipient pairs have found that transplants from younger donors were associated with the same if not better outcomes than older donor pairs [7,11]. However, in many of these studies, this donor-recipient pair would have been excluded based on the extremely young age of this donor (five days old) [7]. Indeed, studies that have included such large age discrepancies reported poorer outcomes with younger donor ages [13]. Interestingly, though the early days post-transplantation exhibited worse allograft function when compared to the others in this series, the patient’s renal function continued to improve and ended up becoming a well-functioning allograft. Though there was no follow-up biopsy for this patient, it is likely based on cases and *in vivo* studies that the glomerular immaturity resolved in the months following transplantation [13-15].

The second case in this series brings up multiple donor-recipient factors that are not often explored. This case had limited data on the donor except for the age, sex, and size of the kidney. There was no donor BMI or BSA available which limits correlation with extant size mismatch literature, and though the age mismatch between this donor-recipient pair was not pronounced like the other cases, the donor was still classified as a pediatric donor which correlated with the suspected immaturity of the glomerular basement membrane. There was also a donor-recipient sex mismatch. Donor-recipient sex mismatch has previously been correlated with poorer outcomes [16,17]. Although, it appears outcomes could be further modulated by age mismatches as well [18]. However, there is insufficient data on the histopathologic correlations with such outcome studies. The findings of persistent lamina densa attenuation could be caused by numerous factors in this patient’s history; however, due to the present age mismatch and lack of other glomerular lesions, it is likely the origin of the lamina densa attenuation is due to a relatively immature glomerulus with basement membranes that are thinner than would be expected in an adult. 

In keeping with previously reported data, this study redemonstrated the previously reported data suggesting age, in particular advanced donor age, were associated with poorer outcomes [7]. The most striking example of these findings was seen in case 3. Though this patient had sufficient renal function immediately after transplantation and stable function for years following transplantation, demise of the renal allograft resulted due to severe damage to the glomerular architecture. The Alport-like changes described in this study have been described before in a patient with previously stable allograft function, but there was no reference to donor-recipient age and/or size mismatch [19]. Prior studies have hypothesized that allografts from older donors tend to portend worse outcomes because of age-related nephrosclerosis which could have played a role in this patient’s allograft; however, further studies would need to be performed to assess this hypothesis [7].

## Conclusions

Taken together, there are numerous factors that should be considered when assessing recipient allograft function in addition to rejection and infection. Though donor-recipient mismatches cannot be modulated in the post-transplant period, knowledge of the mismatch can help guide the interpretation of renal histologic and ultrastructural findings in the renal allograft. Additionally, this study highlights the importance of a thorough workup of acute renal allograft dysfunction, including histologic and ultrastructural analysis in kidney transplant recipients. This study also highlights the importance of further research into the pathophysiologic changes that occur in patients who have a donor-recipient age and/or size mismatch. Though this study is limited by its sample size, the findings are unique and warrant more large-scale exploration to determine the full range of histologic and ultrastructural findings in cases of age and/or size mismatches between donors and recipients. 
